# MicroRNA profiles of MS gray matter lesions identify modulators of the synaptic protein synaptotagmin‐7

**DOI:** 10.1111/bpa.12800

**Published:** 2019-11-17

**Authors:** Lena Fritsche, Sarah Teuber‐Hanselmann, Daniel Soub, Kim Harnisch, Fabian Mairinger, Andreas Junker

**Affiliations:** ^1^ Institute of Neuropathology University Hospital Essen D‐45147 Essen Germany; ^2^ Institute of Pathology University Hospital Essen D‐45147 Essen Germany

**Keywords:** axonal transport disturbance, gray matter lesions, miRNA profiling, microRNA, multiple sclerosis, Synaptotagmin7

## Abstract

We established microRNA (miRNA) profiles in gray and white matter multiple sclerosis (MS) lesions and identified seven miRNAs which were significantly more upregulated in the gray matter lesions. Five of those seven miRNAs, miR‐330‐3p, miR‐4286, miR‐4488, let‐7e‐5p, miR‐432‐5p shared the common target synaptotagmin7 (Syt7). Immunohistochemistry and transcript analyses using nanostring technology revealed a maldistribution of Syt7, with Syt7 accumulation in neuronal soma and decreased expression in axonal structures. This maldistribution could be at least partially explained by an axonal Syt7 transport disturbance. Since Syt7 is a synapse‐associated molecule, this maldistribution could result in impairment of neuronal functions in MS patients. Thus, our results lead to the hypothesis that the overexpression of these five miRNAs in gray matter lesions is a cellular mechanism to reduce further endogenous neuronal Syt7 production. Therefore, miRNAs seem to play an important role as modulators of neuronal structures in MS.

## Introduction

### Cortical lesions in MS are increasing in the course of the disease

In multiple sclerosis (MS), one distinguishes lesions of the white (WM) and gray (GM) matter. Depending on their localization within the cortex, GM lesions are subdivided into leucocortical (type I), intracortical (type II) and subpial (type III) lesions 11. In the course of the disease, the cortical lesion area increases disproportionately compared to WM lesions 2, 42. Thus, in secondary chronic progressive forms, the GM lesion area reaches more than half of the area of the WM lesions 42. As in WM lesions, the cortical lesions show a pronounced axonal reduction with an additional mild reduction in neuronal density 86, 87, 97. Cortical lesions exhibit inflammatory changes in their early phase similar to WM lesions 51, although the extent of inflammation is always less. Chronic GM lesions show virtually no inflammatory infiltrates 12. Nonetheless, the effects of inflammatory alterations on neurophysiological processes are not yet sufficiently understood.

### Pathophysiology of cortical lesions

In attempts to gain a better insight into the pathophysiology of the cortical lesions, most experimental approaches have focused on inflammatory demyelination 43, 59, and the importance of pro‐inflammatory cytokines and anti‐myelin protein antibodies on the development of MS‐like lesions have been demonstrated in animal models. Inflammatory alterations of GM lesions are associated with synaptic changes, which are principally reversible and therefore represent an interesting therapeutic target 55. Recently, it was shown that inflammation‐triggered neuronal changes contribute to phagocyte‐mediated synaptic loss in an animal model 19. Cortical lesions may therefore play an important role in neurocognitive changes in patients 15, 73.

### Epigenetic influence of miRNAs on the pathophysiology of MS

Epigenetic mechanisms, such as miRNA regulation, might have a significant impact on the pathophysiology of GM lesions. Mature miRNAs are single‐stranded RNA molecules with an average length of about 22 nucleotides. They are released from larger hairpin‐folded RNA precursors, known as pre‐miRNAs, by processing via the enzyme Dicer (for review 6). Mature miRNAs bind to specific sequences in the 3′ UTR of their target mRNAs and thus influence gene expression, either by inhibiting translation or by degrading the transcripts of the target mRNA^24^, ^99^. Remarkably, one single miRNA can bind to several hundred target genes 5, 79. It is estimated that the expression of more than one third of all mammalian genes is regulated by miRNAs 46. In recent years, numerous papers have been published on miRNA alterations in blood and CSF of MS patients, but only little is known about miRNA regulation in parenchyma of the central nervous system; most of those few studies focused on WM lesions 22, 32, 37, 40, 41, 64, 70, 71, 84. In MS, altered miRNAs show effects on differentiation and activation of T cells, myelination and gene expression in astrocytes and microglia cells 36. For example, miR‐155 37, which is prominently upregulated in active WM MS lesions, regulates transcription factors such as c‐Maf, cytokines and signaling proteins (for review see 25). MiR‐219, which is significantly downregulated in chronic WM lesions, (37 and current study), plays an important role in the differentiation of oligodendrocytes 20, 100. In a mouse model with deletion of the miRNA processing enzyme Dicer, the absence of miR‐219 led to activation of demyelinating processes in mature oligodendrocytes, oxidative damage, lipid accumulation, inflammation, astrocytosis, microgliosis and ultimately neuronal degeneration as well as axonal damage 82. To our knowledge, only two studies have to date investigated miRNA regulation in the GM in MS or MS‐like lesions. The first one studied miRNA profiles in spinal lesions of experimental autoimmune encephalomyelitis (EAE) animals and identified a total of 14 miRNAs that were differentially regulated in murine neurons 39. The second study investigated neuronal miRNAs in demyelinated human hippocampi and demonstrated elevated levels of neuronal miRNA‐124 and its association with reduction of AMPA receptors 21.

So far, no research has *systematically described miRNA regulation in GM lesions in MS*. Thus, the aim of the present study was to determine whether epigenetic mechanisms, such as regulation by miRNAs, influence gene expression in cortical lesions. We were particularly interested in altered expression levels of neuronal genes that might display functional relevance in the context of synaptic activity.

## Materials and Methods

### Human brain tissue

The investigations were performed on formalin‐fixed and paraffin‐embedded (FFPE) autopsy tissue from 16 autopsied MS patients, as well as biopsy material from 8 MS patients and from 12 age‐matched autopsy controls (Supplementary Table S1). All of the autopsied patients had suffered from long‐term chronic MS.

The material from the autopsied MS patients was obtained from the Netherlands Brain Bank (NBB), and the diagnosis was confirmed by Andreas Junker. The examined tissue originates from the parietal and frontal cortex. The control tissues were obtained from the archives of the Institute of Neuropathology of the University Hospital Essen, where the diagnoses had been made. The clinical history of the patients was evaluated for the study. The study was approved by the ethics committee of the Ethics Commission of the University of Duisburg‐Essen (reference: 17‐7670‐BO). All of the investigations were performed in compliance with relevant laws and institutional guidelines and were carried out in accordance with the Declaration of Helsinki of 1964, revised in 2013.

### Histology and Immunohistochemistry

All of the investigations were performed on 1 μm tissue sections. In addition to standard staining with hematoxylin‐eosin (HE) (not shown) or Klüver Barrera, immunohistochemical staining was performed with antibodies against Syt7, NF, Syn, CNP, MBP, Olig2, NeuN, GFAP, SMI31 and SMI32 according to standard procedures. Pretreatments and antibody dilutions were carried out as described in Supplementary Table S2.

In brief, as described previously 34, the endogenous peroxidase activity was first blocked by incubating the sections in 3% H_2_O_2_ in PBS. This was followed by a blocking step with 10% fetal calf serum in PBS for 10 minutes at room temperature, followed by incubation with the primary antibody for 1 h at room temperature. The sections were then incubated with the secondary antibody (fluorescence or biotin labeled antibody). Finally, the conventional immunohistochemical staining was developed with 3,3′‐diaminobenzidine (DAB). Cell nucleus counterstaining was performed with hematoxylin (for conventional staining) or with 4′,6‐diamidino‐2‐phenylindole (DAPI) for fluorescence staining. Some sections were stained using the DAKO Autostainer Plus^®^. In these cases, the ZytoChemPlus^®^ HRP Polymer System (Mouse/Rabbit) (REF:POLHRP‐100) was used for detection. In addition, immunofluorescence double staining was performed with Syt7 + NF, Syt7 + Syn, Syt7 + CNP, Syt7 + NeuN, Syt7 + Olig2 and Syt7 + GFAP (Supplementary Table S2 and Supplementary Fig. S1). The anti‐SMI32 staining gave no evaluable results with the autopsy tissue; data not shown.

The stained sections were first digitized using a Leica slide scanner. From the scanned files, five areas with an edge length of 500 µm were extracted from all regions of interest and analyzed using Image J^®^ 75. After adjusting hue, saturation and brightness, the “color threshold” was adjusted so that colored particles or colored areas could be determined using the “Analyze Particles” function 91. In WM, centers of chronically inactive demyelinated lesions and normal appearing areas were analyzed in each slice, if present. In GM, subpial lesion areas, normal appearing subpial areas, leucocortical lesion areas and normal appearing leucocortical areas were analyzed in each slice, if present. In addition, corresponding control tissue from healthy controls was investigated.

### Luciferase‐Assays for analyzing miRNA targeting

Oligonucleotides of 50 to 60 base pair length (Metabion, Martinsried, Germany) containing specific miRNA‐binding sites (sequences shown in Supplementary Table S3) were cloned into the 3′ untranslated region (UTR) of luciferase in a reporter plasmid (pMIR‐REPORT^TM^ miRNA Expression Reporter Vector System Ambion/Applied Biosystems/ThermoFisher Scientific) as previously described 37. Pre‐miRs^TM^ (Ambion/Applied Biosystems/ThermoFisher Scientific) were transfected into HeLa cells with Lipofectamin2000 (Invitrogen, Karlsruhe, Germany) along with the luciferase plasmid containing the predicted binding site of the respective miRNA. A control plasmid coding for beta‐galactosidase without any known miRNA‐binding site in its 3′UTR was used for normalization of the luciferase signal; 200 ng of each plasmid and 25 nmol of pre‐miRNA were applied to transfect 8x10^4^ HeLa cells. Luciferase and beta‐galactosidase were measured 24 h after triple transfection (see above) using the Dual Light Luciferase Assay from Applied Biosystems/ThermoFisher Scientific and the Infinite 200 from Tecan.

### Nanostring

Nanostring analyses were carried out as previously described 34, 92. In brief, relevant areas were extracted by macrodissection from 5 µm paraffin sections that had previously been mounted on foil‐coated slides (MS patients and controls). Total RNA was obtained using the miRNeasy FFPE kit (Qiagen, Hilden, Germany). From each RNA sample, the relevant miRNAs and transcripts (Syt7, GAPDH, B2M) were quantified using nanostring technology. Therefore, the total amount of RNA (100 ng) from each sample was hybridized overnight and evaluated using a digital analyzer (NanoString, Seattle, WA, USA). A specially prepared multiplex probe library was used, each with two sequence‐specific probes. The capture probe (35–50 bp) was coupled to biotin, and the reporter probe (35–50 bp) to a color code.

### Cell culture

SH‐SY5Y cells (ATCC, Wesel, Germany) (28th passage) were cultured according to standard culture conditions (DMEM/F12, 10% fetal calf serum – PAN Biotech, Aidenbach, Germany). Pre‐miRs^TM^ (Ambion/Applied Biosystems/ThermoFisher Scientific) or a scrambled Pre‐miR^TM^ control—Pre‐miR control‐1 (Ambion/Applied Biosystems/ThermoFisher Scientific)—was transfected into these cells with Lipofectamin2000 (Invitrogen, Karlsruhe, Germany). After 24 h of incubation, cells were harvested and total RNA was obtained using the miRNeasy kit (Qiagen, Hilden, Germany).

### Real‐time PCR

Random hexamer primers (High‐Capacity cDNA Reverse Transcription Kit, Applied Biosystems, Darmstadt, Germany) were used for cDNA synthesis. The qPCR was performed on the ABI 7500 Fast Real‐Time System (Applied Biosystems) using the TaqMan Universal PCR Master Mix (Applied Biosystems). For the analysis of Syt7 transcript, a Taqman assay (Applied Biosystems) was used and GAPDH (Applied Biosystems) was used for normalization in the delta CT method 63.

### Statistical analysis

GraphPad Prism 5.0 was used for statistical analysis and evaluation. The Mann–Whitney U test was used to compare independent groups. The correlation between groups was calculated as Pearson's *r*. One‐way ANOVA for non‐parametric data was utilized to compare more than one group with each other. A *P*‐value of <0.05 was considered to be statistically significant and <0.01 as highly significant.

## Results

### miRNA pattern of GM and WM lesions

In a previous study, the last author of the present study (AJ) had already detected dysregulated miRNAs in active and chronic‐inactive WM lesions 37. The present study focused on dysregulated miRNAs in cortical lesions.

miRNA profiles were investigated in 18 different post‐mortem MS brains and 12 different age‐matched cerebral autopsies without any recognizable neuropathological changes. In total, miRNA profiles of 14 gray matter lesions [seven subpial lesions (type III) and seven leucocortical lesions (type I)] from different patients were investigated. Since purely intracortical (type II) lesions are usually very small and have very low RNA concentrations, they were neglected here. In addition, seven chronically inactive WM lesions from different MS patients were examined. Normal appearing WM (NAWM) was investigated as well. Subpial and leucocortical areas of normal GM, as well as normal WM from age‐matched inconspicuous cerebral autopsies (healthy controls) served as control tissues.

There was high agreement of the miRNA profiles concerning WM lesions between the present and the previous study 37. Nonetheless, probably due to the highly sensitive nanostring technology 90, the current study uncovered more regulated miRNAs than the previous one (Supplementary Table S4).

Overall, compared to healthy controls we found an at least twofold increase of the expression levels of 20 miRNAs in subpial (type III) lesions (Table 1), 15 miRNAs in leucocortical (type I) lesions (Table 1) and 36 miRNAs in WM lesions (Supplementary Table S4), while the expression levels of 11 miRNAs in subpial lesions, 13 miRNAs in leucocortical lesions and 23 miRNAs in WM lesions were reduced by at least half compared to controls (Table 1 and Supplementary Table S4).

**Table 1:** The miRNA‐expression in gray matter MS lesions

miRNAs upregulated in lesions†	Percent surrogate housekeeping gene‡ in lesions	Fold regulation in lesions compared to normal brain GM§	miRNAs downregulated in lesions†	Percent surrogate housekeeping gene‡ in lesions	Fold regulation in lesions compared to normal brain GM§
*miRNA profiles in subpial lesions*					
miR‐574‐5p	19.3	29.1**	miR‐219a‐5p	6.5	0.12**
miR‐4488	2.9	12.8*	miR‐144‐3p	5.1	0.21*
miR‐1285‐5p	36.9	12.1*	miR‐574‐3p	0.2	0.23*
miR‐320e	15.3	10.0*	miR‐219a‐2‐3p	7.9	0.24**
miR‐2682‐5p	1.9	9.3*	miR‐194‐5p	0.4	0.27*
miR‐548ah‐5p	3.4	8.1*	miR‐32‐5p	2.6	0.43*
miR‐122‐5p	17.7	8.1**	miR‐660‐5p	0.6	0.44*
miR‐888‐5p	5.9	7.6**	miR‐190a‐5p	1.3	0.44*
miR‐3065‐5p	9.5	6.6*	miR‐133a‐3p	1.1	0.45*
miR‐4286	10075.5	6.6**	miR‐328‐3p	1.5	0.49*
miR‐3144‐3p	4.6	3.9*	miR‐151a‐5p	3.6	0.50*
miR‐1290	13.1	3.8*			
miR‐432‐5p	13.8	3.0**			
miR‐1260a	50.2	2.6*			
miR‐378i	5.5	2.5*			
miR‐139‐3p	14.5	2.3**			
miR‐1972	23.2	2.2*			
miR‐1180‐3p	91.9	2.0*			
miR‐612	7.5	2.0*			
let‐7e‐5p	77.9	2.0**			
*miRNA profiles in leucocortical lesions*					
miR‐574‐5p	38.7	75.4**	miR‐574‐3p	0.1	0.08*
miR‐4488	2.6	25.2**	miR‐219a‐5p	13.8	0.09**
miR‐1285‐5p	70.7	24.6**	miR‐219a‐2‐3p	15.2	0.09**
miR‐888‐5p	5.7	11.2**	miR‐194‐5p	0.4	0.20**
miR‐3065‐5p	18.0	11.0*	miR‐32‐5p	2.4	0.25**
miR‐548ah‐5p	2.3	8.7*	miR‐151a‐5p	3.7	0.28*
miR‐4286	10207.9	7.1**	miR‐197‐3p	1.1	0.34*
miR‐1972	52.8	6.3*	miR‐181b‐5p +	3.9	0.36*
miR‐1260a	78.0	6.1**	miR‐181d‐5p		
miR‐612	9.6	5.8**	miR‐33a‐5p	1.0	0.37*
miR‐3144‐3p	9.1	5.1*	miR‐190a‐5p	1.2	0.40*
miR‐139‐3p	14.0	2.9**	miR‐181a‐3p	1.9	0.42*
miR‐432‐5p	11.9	2.4*	miR‐26b‐5p	16.0	0.47*
miR‐1180‐3p	84.4	2.4*			
let‐7e‐5p	76.3	2.1**			

^†^ The miRNAs listed were significantly upregulated by at least twofold (**P < 0.01,*P < 0.05; U‐test) in GM subpial‐ or leucocortical lesions compared to normal brain.

^‡^ Surrogate housekeeping gene: the median of the 36 most abundant miRNAs (median copy number > 800).

^§^ Seven subpial lesions, seven leucocortical lesions and seven control subpial regions and seven control leucocortical regions were examined.

In addition, we found 4 upregulated and 19 downregulated miRNAs in the NAWM of MS brains compared to the WM of healthy controls (Supplementary Table S4).

### Six miRNAs show stronger upregulation in GM lesions than in WM lesions

**Figure 1:**
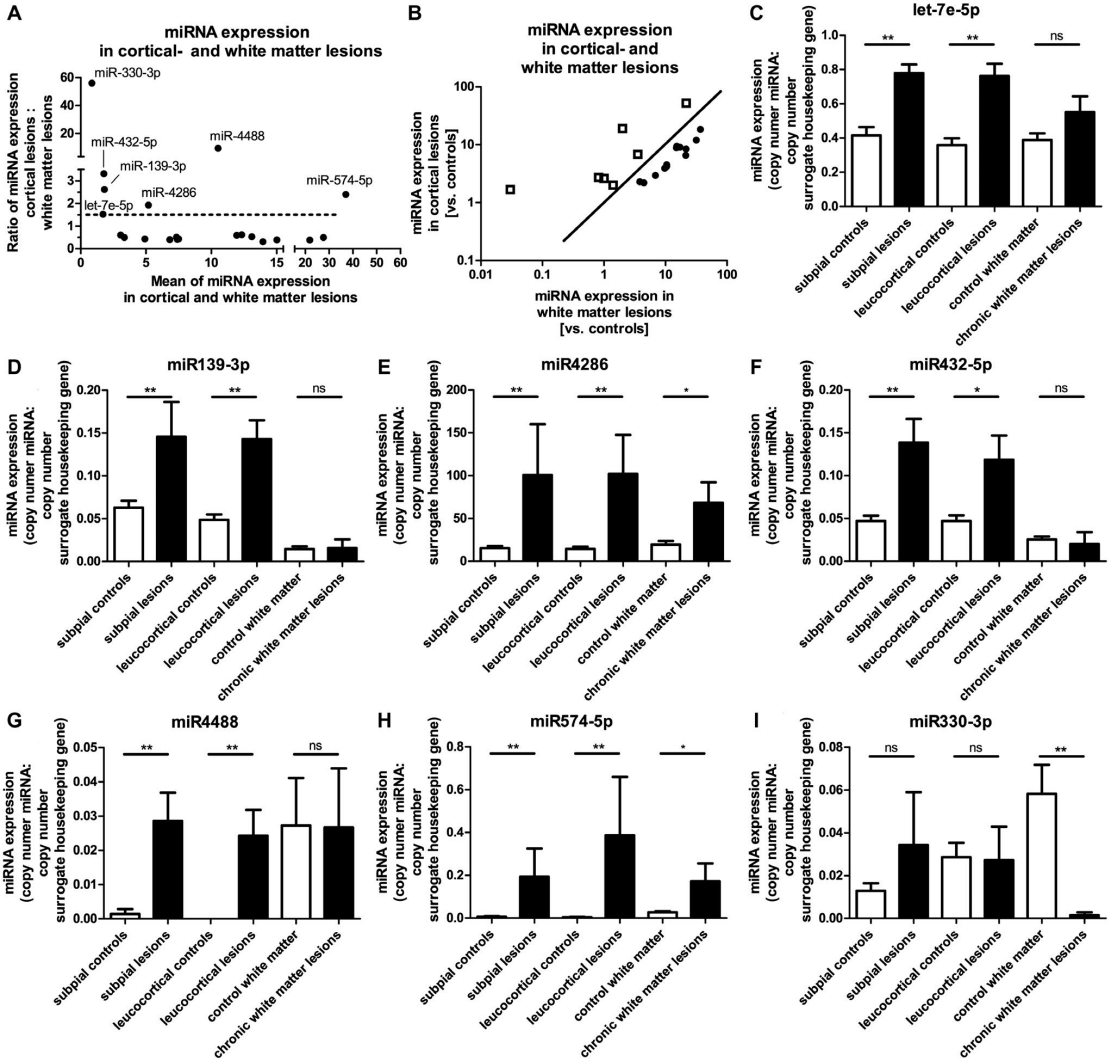
*miRNAs with higher upregulation in GM lesions than in WM lesions*. Figure 1 summarizes all miRNAs that were elevated at least twofold in cortical lesions (either subpial or leucocortical), and that were less upregulated, unregulated or downregulated in WM lesions. To illustrate the miRNAs, which are regulated differently in WM and GM, the ratio of miRNA expression of cortical lesions vs. white matte lesions (y‐axis) vs. the mean value of miRNA expression in these areas (x‐axis) is shown in **A**. The miRNAs chosen for further experiments were all regulated at least 1.5 times higher in the GM than in the WM. (dotted line). In B a different kind of representation of this ratio was chosen, showing the cortical miRNA expression vs. the miRNA expression in the white matter. The squares in **B** represent the miRNAs that are higher regulated in GM lesions than in WM lesions (dots). These were miRNAs let‐7e‐5p (**C**), miR‐139‐3p (**D**), miR‐4286 (**E**), miR‐432‐5p (**F**), miR‐4488 (**G**), miR‐574‐5p (**H**) and miR‐330‐3p (**I**), whose expressions were shown in different MS areas and the corresponding healthy controls.

Most miRNAs that appeared to be upregulated or downregulated in GM lesions were regulated in the same manner in WM lesions. In detail, 13 of the upregulated miRNAs in GM lesions (subpial and/or leucocortical) were regulated in the same way in WM lesions. Twelve miRNAs that were downregulated in GM lesions also appeared reduced in WM lesions. Figure 1 highlights the upregulated miRNAs in the GM lesions (Figure 1A,B).

Increased upregulation of miRNAs in GM lesions compared to WM lesions indicates that these miRNAs might be *neuronal miRNAs*. In neurons, a compartmentalization of miRNAs is observed; some miRNAs are mainly present in the cell soma, while others are also found in axons or dendrites 93. Therefore, increased miRNA expression in GM lesions but not or to a lesser extent in WM lesions indicates a localization in the neuronal cell soma. Therefore, we focused on seven miRNAs that were upregulated to levels at least 1.5 times higher in GM lesions than in WM lesions. Of these seven miRNAs, four (let‐7e‐5p, miR‐139‐3p, miR‐432‐5p, miR‐4488) were not significantly regulated in WM lesions compared to healthy controls (Figure 1C,D,F,G), indicating a strict localization in neuronal soma. The two remaining miRNAs (miR‐4286 and miR‐574‐5p, Figure 1E,H, respectively) showed significant upregulation in WM lesions compared to healthy controls, but this upregulation was significantly lower than it was in GM lesions (compared to healthy controls) (miR‐4286: WM lesions 3.5‐fold* upregulation; GM lesions (subpial): 6.6‐fold** upregulation; GM lesions (leucocortical): 7.1‐fold** upregulation; miR‐574‐5p: WM lesions 21.8‐fold* upregulation; GM lesions (subpial): 29.1‐fold** upregulation; GM lesions (leucocortical): 75.4‐fold** upregulation). A significant increase of miRNA expression in WM lesions as demonstrated in miR‐4286 (Figure 1E) and miR‐574‐5p (Figure 1H) may indicate an additional expression in reactive astrocytes.

One further miRNA, miR‐330‐3p, did not show significantly elevated (but stable) expression in GM lesions, but it was anyhow significantly downregulated in WM lesions compared to controls (Figure 1I). A reduction of miRNA abundance due to a reduced number of axons in white matter lesions seems unlikely due to stable expression in GM lesions where the axonal scaffold is also reduced. This led to the hypothesis that miR‐330‐3p is expressed in oligodendrocytes. Since mature oligodendrocytes are significantly reduced in number within WM lesions, this loss of oligodendrocytes might have resulted in decreased miRNA expression.

Interestingly, the occurrence in neurons has been described for the miRNAs miR‐330 14 and miR574 49 and some miRNAs of the let‐7 family 58, 60, 74, 94, 101.

After identification of these above named miRNAs, we performed an *in‐silico* analysis with Targetscan^®^ 30, 46 to determine their potential targets. Among these, we found some genes mainly expressed in neurons.

### Syt7 is a shared target of miRNAs specifically upregulated in GM lesions

**Figure 2:**
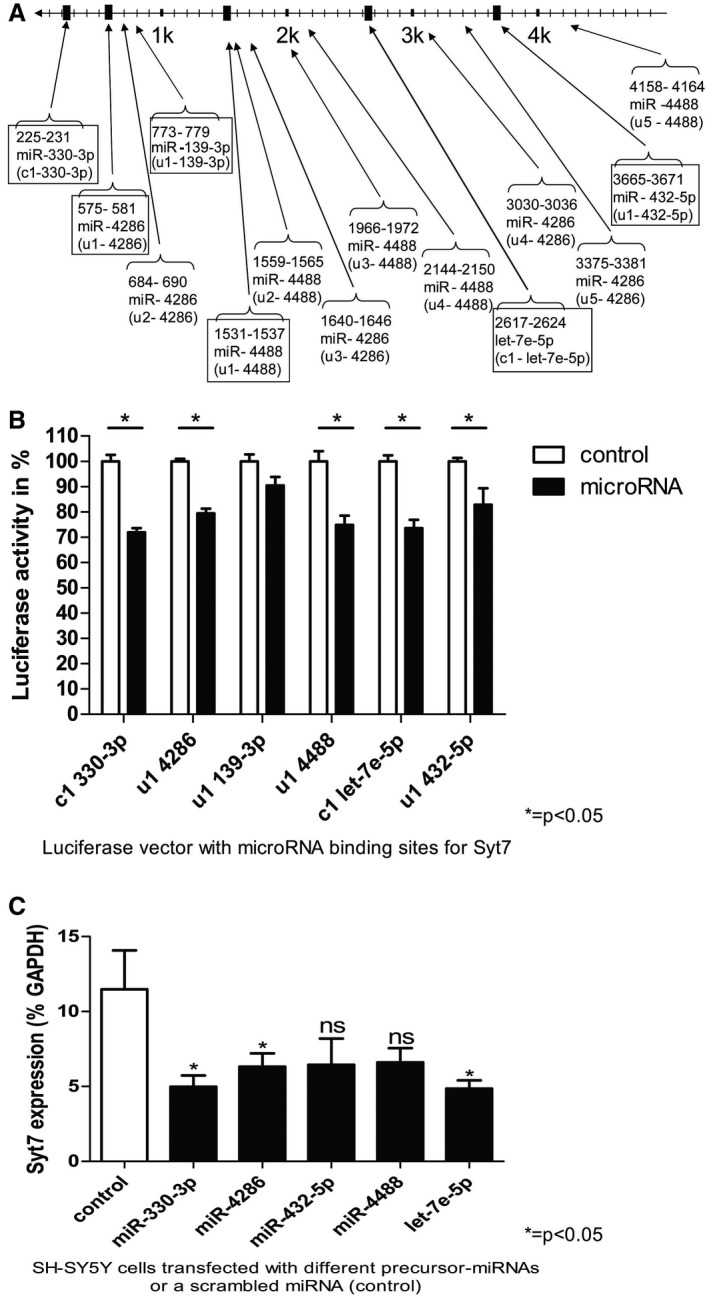
*microRNAs target the 3′ UTR of Syt7*. **A.** The 3′ untranslated region (UTR) of Syt7 with the *in silico*‐predicted binding sites of miRNAs, which were more upregulated in GM lesions than in WM lesions in MS. miRNA‐binding sites of human Syt7 3′UTR ENST00000263846.4 (length 4911 BP) were calculated with Targetscan^®^ human (release 7.1: June, 2016). For two miRNAs (miR‐4286, miR‐4488), several binding sites in Syt7‐3′UTR were predicted; the binding site located farthest in the 5′ direction was examined (binding sites in closed boxes). **B**. Luciferase activity was studied in HeLa cells transfected with the indicated luciferase vector with or without its corresponding miRNAs. A control vector with beta‐galactosidase was used for normalization. The measured luciferase activity was divided by the beta‐galactosidase activity. Luciferase measurements were performed in quadruplicates in each individual experiment. The SEM of three to four experiments is shown. *P* values were determined by Mann–Whitney U tests. (*P < 0.05) Details of the luciferase reporter vector used can be found in Supplementary Table S3. Therefore, “c” indicates a highly conserved binding site and “u” indicates a less conserved binding site. **C.** SH‐SY5Y cells were transfected with miRNAs which reached a significant reduction of luciferase activity in (**B**). After 24 h incubation time, Syt7 expression was determined using real‐time PCR. GAPDH was used as a reference housekeeping gene and the Syt7 expression is depicted in % GAPDH expression. All five miRNAs showed a reduction of app. 60‐70% Syt7 expression. Mean expression of Syt7 of three independent experiments is shown together with the SEM. Three replicas are not sufficient to achieve significant P‐values, which are therefore not shown.

Our *in‐silico* analysis converged on the neuronal gene Syt7. Syt7 shows potential binding sites in its 3′ untranslated region (UTR) for five out of six above named miRNAs. For two miRNAs (miR‐4286 and miR‐4488), several binding sites were predicted. Furthermore, Syt7 reveals a potential binding site for miR‐330‐3p, which was particularly decreased in WM lesions compared to GM lesions (Figure 2A).

To verify the interactions of miRNAs and their predicted binding sites, we performed luciferase assays. For those miRNAs with multiple binding sites, the one located farthest in the 5′ direction was examined. Five miRNAs—miR‐330‐3p, miR‐4286, miR‐4488, miR‐let‐7e‐5p, and miR‐432‐5p—showed significant interactions with their predicted Syt7‐binding sites, resulting in a decreased luciferase activity of up to 30%. Only miR‐139‐3p did not show significant interactions with its predicted binding site (Figure 2B).

In fact, *in vitro* analyses confirmed these interactions between miRNAs and Syt7. We transfected each single of the five miRNAs into SH‐SY5Y cells. The amount of Syt7 expression was measured via qPCR 24 h later (Figure 2C). miRNA transfection resulted in significantly downregulated Syt7 levels by about 50% (miR‐330‐3p, miR‐4286 and microRNA let‐7e‐5p).

### Syt7 is expressed in neurons, axons and oligodendrocytes

Double immunofluorescence stainings verified the diverse cellular origin of Syt7. The neuronal origin of Syt7 was proven by double staining against synaptophysin and Syt7 (Supplementary Fig. S1B) or NeuN and Syt7 (Supplementary Fig. S1D), the axonal origin by double staining against NF and Syt7 (Supplementary Fig. S1A) and the oligodendroglial origin by double staining against Olig2 and Syt7 (Supplementary Fig. S1E) or CNP and Syt7 (Supplementary Fig. S1C). Processes of reactive astrocytes showed no Syt7 immune‐positivity (staining against Syt7 and GFAP, Supplementary Fig. S1F).

**Figure 3:**
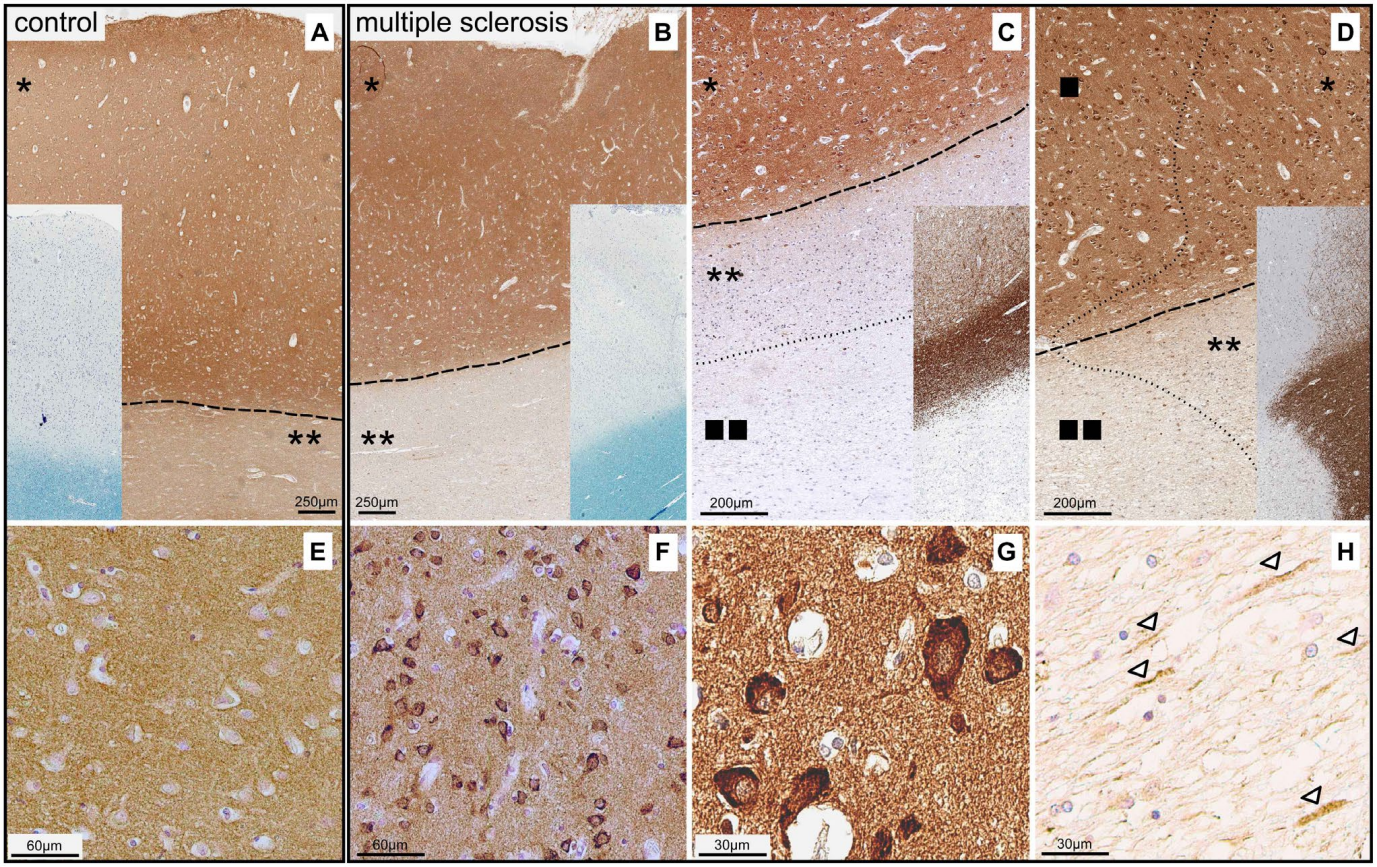
*Accumulation of Syt7‐protein in neuronal soma and individual axons of cortical lesions and NAGM in MS*. **A**. The immunohistochemical examination of Syt7 in healthy controls revealed a significantly more intense staining in the cortex (*) than in the WM (**) (The border between cortex and WM is marked with a dashed line). Remarkably, normal appearing white matter (NAWM) in MS brains (B**) showed an overall less intense staining than healthy control WM (A**), while myelin sheaths are not reduced in MS cases compared to healthy controls (Klüver staining—inset in **A** and **B**). In demyelinated areas (verifiable as the absence of the MBP colored myelin sheaths—inset in **C** and **D**), there was an even stronger reduction of Syt7 (**C, D** ■■); the border between normal appearing tissue (* or **) and lesion area (■ or ■■) is marked with dotted lines. In the gray matter lesions as opposed to healthy cortical controls (Figure 3E), we observed Syt7 accumulation in the soma of the neurons (**D**■ and at higher magnification in **F** and **G**). Accumulation in neuronal cell soma was also detectable outside the lesions, ie, in NAGM, often in the vicinity of a subcortical lesion in the WM (D*). In WM lesions, but also in NAWM, adjacent to lesions, single axons with Syt7 accumulation were found (**H**, arrowheads).

The immunohistochemical staining against Syt7 highlighted a significantly stronger immunoreactivity in the cortex (Figure 3A*) than in WM (Figure 3A**), which implies that Syt7 is mainly of neuronal origin. Moreover, we observed accumulation of Syt7 in the soma of neurons in GM lesions (Figure 3D■ and at higher magnification in Figure 3F,G) as well as in normal appearing gray matter, often in the vicinity of a subcortical lesion (Figure 3D*). In fact, this Syt7 accumulation in neuronal soma was not recognized in the corresponding gray matter from healthy controls (Figure 3E).

Nonetheless, Syt7 was also expressed in axons in white matter. Interestingly, WM from MS brains exhibited a lower overall staining intensity than WM from healthy control tissue, both in NAWM (Figure 3B‐D**) and in WM lesions (Figure 3C,D■■). This indicates that reduction of Syt7 is also present in WM areas without myelin loss, ie, histologically non‐damaged white matter. In demyelinated areas (Figure 3C,D■■), we found even fewer Syt7 positive axonal structures than in NAWM in the direct vicinity of the lesion (Figure 3C,D**). Interestingly, individual axons with Syt7 accumulation were seen in WM lesions, but also in NAWM, adjacent to lesions (Figure 3H). Since Syt7 staining of apparently normal cortex in MS and in healthy controls reached almost identical staining intensities, accompanied by equal staining affinity of myelin sheaths in MS and control tissues (Figure 3A,B, each inset), a fixation‐dependent staining artifact is virtually ruled out.

**Figure 4:**
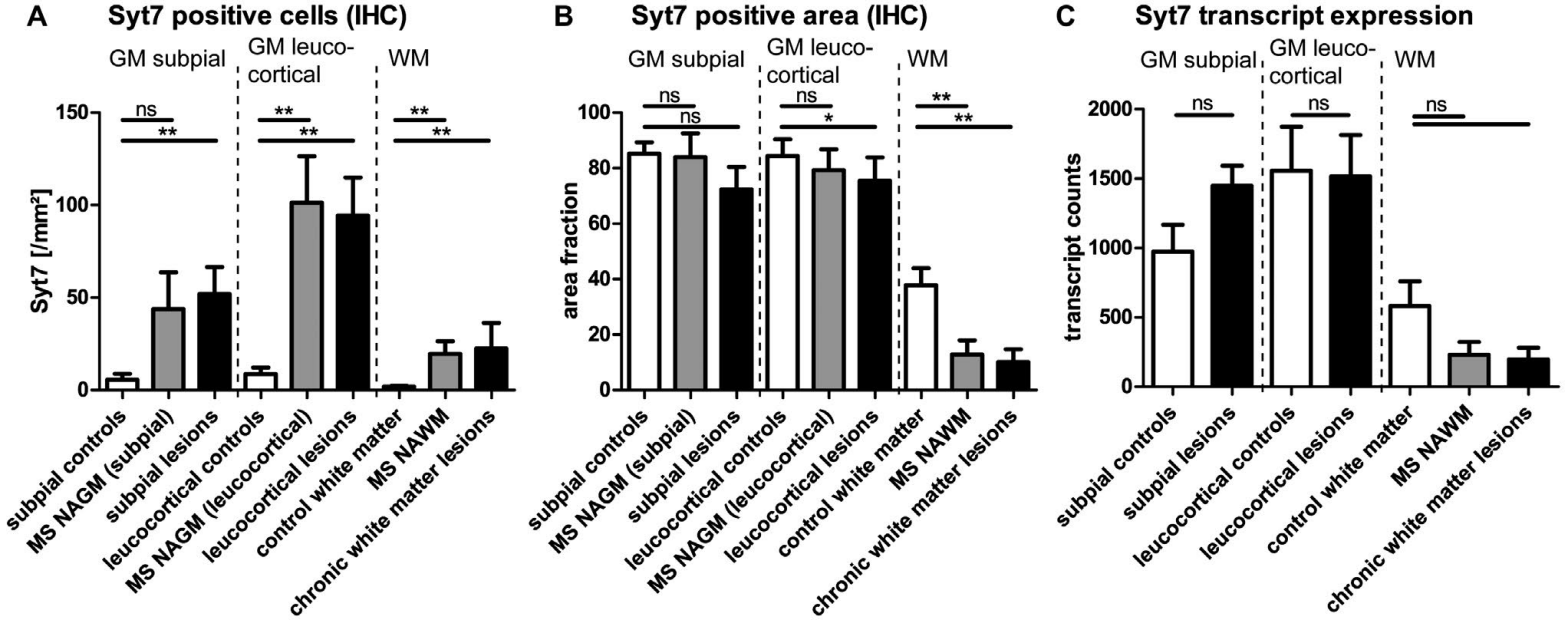
*Syt7 protein abundance and transcript expression in MS‐tissue*. **A**. A larger number of Syt7 positive cells was detectable in subpial lesions of MS patients compared to subpial areas of healthy controls. In leucocortical areas, considerably more Syt7 positive cells were detected than in corresponding leucocortical areas of healthy controls. In NAWM and in chronic WM lesions of MS, more Syt7 positive cells were recognizable than in control WM. **B**. The area stained with Syt7 (resembling stained cell processes) in leukocortical lesions, in NAWM and in chronic WM lesions was decreased compared to control GM/WM. **C**. Syt7 transcripts did not differ significantly, but showed a trend toward fewer Syt7 transcripts in NAWM and chronic white matter lesions. Glyceraldehyde 3‐phosphate dehydrogenase (GAPDH) and beta‐2 microglobulin (B2M) served as housekeeping genes for comparison. The number of Syt7 positive cells and the percentage area staining for by Syt7 were determined by ImageJ^®^ (**A, B**). The amount of transcripts was quantified using nanostring technology (**C**). Mean values + SEM are given. *P < 0.05; **P < 0.01. The point of reference for the statistical analysis (Mann–Whitney rank sum test) was always the corresponding control tissue of the white or GM of healthy controls (**A, B**, subpial controls: n = 12, MS NAGM (subpial): n = 8, subpial lesions: n = 15, leucocortical controls: n = 12, MS NAGM (leucocortical): n = 10, leucocortical lesions: n = 12, control WM: n = 12, MS NAWM: n = 13, chronic WM lesions: n = 13; **C**, subpial controls: n = 8, subpial lesions: n = 11, leucocortical controls: n = 8, leucocortical lesions: n = 9, control WM: n = 8, MS NAWM: n = 7, chronic WM lesions: n = 9).

Quantification of SYT7 protein using ImageJ^®^ graphics software underlined the finding of Syt7 accumulation in neuronal cell bodies in both GM lesions and NAGM in comparison to healthy control tissue (Figure 4A; *P* < 0.01 and Supplementary Fig. S2). Interestingly, there was an increased cell count in WM areas as well. This might be explained by a larger amount of Syt7 expressing oligodendrocytes, which are—despite lower numbers of mature oligodendrocytes in MS tissues in general—far more often in white matter of MS patients (WML and NAWM) than in healthy controls (Figure 4A; *P* < 0.01 and Supplementary Fig. S2).

Moreover, the obvious decrease of Syt7 protein in axons reached significance, especially in WM lesions (Figure 4B, *P* < 0.01), but also in leucocortical lesions (Figure 4B, *P* < 0.05) compared to healthy controls. Additionally, a significant reduction of Syt7 was evident in the NAWM compared to healthy controls (Figure 4B, *P* < 0.01).

Interestingly, the amount of Syt7 transcripts (evaluated using nanostring technologies) showed no significant differences in gray and white matter lesions compared to control tissues (Figure 4C, n.s.), although a tendency toward fewer Syt7 transcripts in NAWM and WM lesions was observed.

The accumulation of Syt7 in neuronal cell bodies and some neurites, coupled with a widespread reduction of Syt7 in WM, even independent of MS lesions, indicates an overall disturbance of the distribution of Syt7 in MS brains.

### A transport disturbance of Syt7 is the potential reason for neuronal accumulation and axonal rarefication of Syt7

**Figure 5:**
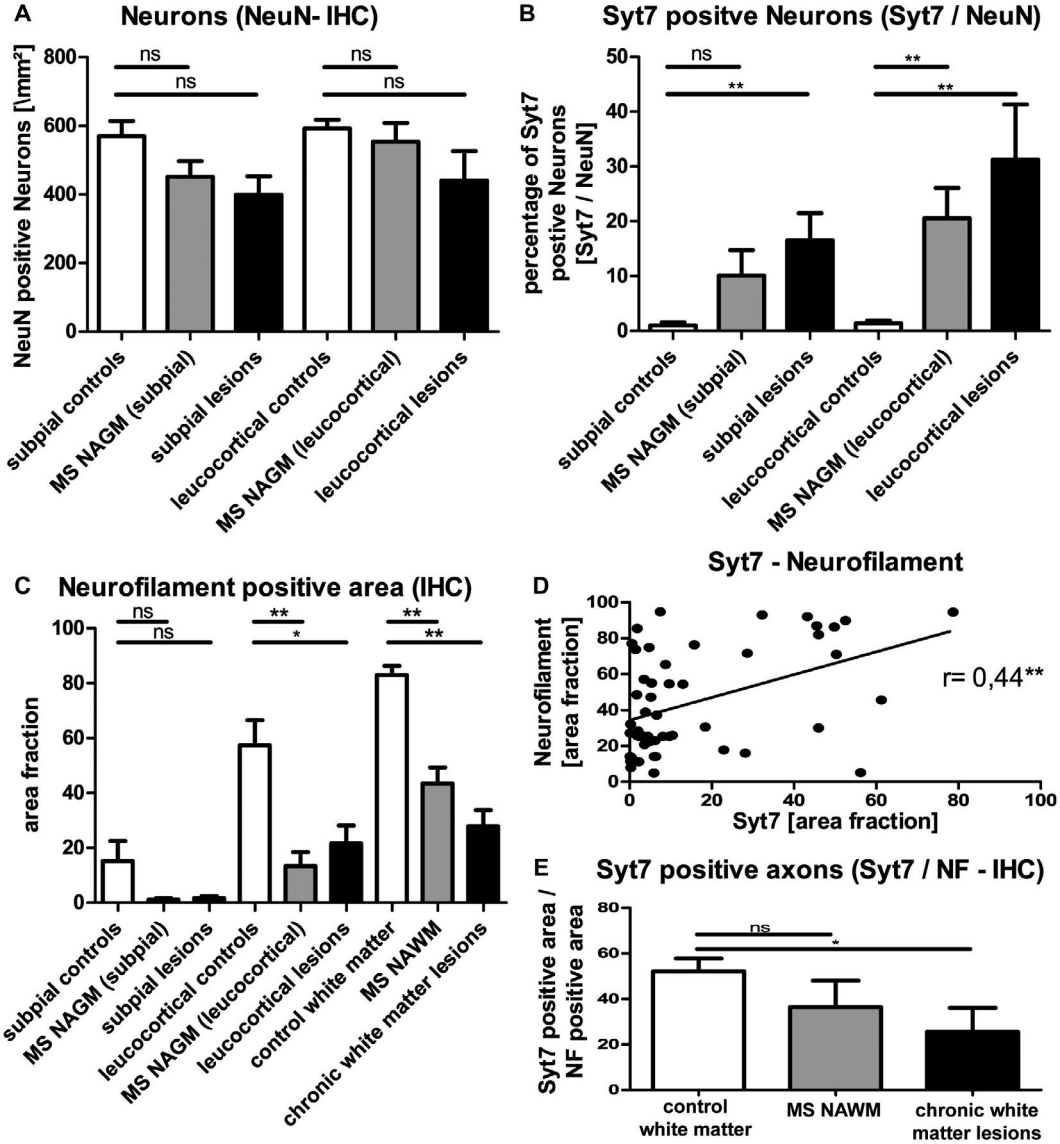
*Neuronal accumulation and axonal rarefaction of Syt7*. Software‐assisted (ImageJ^®^) quantification of immunopositive cells showed a minor decrease of neurons in subpial and leucocortical cortex in MS (**A**), not limited to lesion areas, but also in the so‐called normal appearing gray matter (NAGM). The number of Syt7 positive neurons (**B**) was increased in NAGM (significantly in leucocortical areas) as well as in subpial and leucocortical lesions. In the software‐assisted (ImageJ^®^) analysis of the area covered by neurofilament (**C**), a significant reduction was found in axonal density in leucocortical lesions and also in normal appearing gray (leucocortical) and white matter in MS tissue. The Syt7‐stained area correlates well with the neurofilament stained area (**D**). However, the quotient of Syt7 to neurofilament shows a more pronounced, but non‐significant reduction of the Syt7 stained area in NAWM and significant reduction in chronic white matter lesions than one might expect from the reduction of axonal density alone (**E**), indicating a possible axonal transport disorder of Syt7. Shown are mean values + SEM. *P < 0.05; **P < 0.01. Pearson correlation coefficient (**D**). The point of reference for the statistical analysis (Mann–Whitney rank sum test) was always the corresponding control tissue of the white or GM of healthy controls (subpial controls: n = 12, MS NAGM (subpial): n = 8, subpial lesions: n = 15, leucocortical controls: n = 12, MS NAGM (leucocortical): n = 10, leucocortical lesions: n = 12, control WM: n = 12, MS NAWM: n = 13, chronic WM lesions: n = 13.

In order to evaluate the extent of Syt7 accumulation in neuronal soma, we quantified Syt7‐positive neuronal somas in relation to the total number of neurons (quantified by staining against the neuronal marker NeuN). While the number of neurons in the cortex of MS patients (GM lesions and NAGM) was not significantly reduced (Figure 5A, n.s.), the percentage of Syt7 positive neurons (Syt7 positive neurons / NeuN positive neurons) was significantly higher in MS lesions than in healthy controls (Figure 5B; *P* < 0.01).

Axonal damage can be determined by comparing axonal densities in diseased and non‐diseased tissues 65, 77. Axonal density is represented by neurofilament staining with a pan‐neurofilament marker (Figure 5C). Notably, there was a reduction of axonal density in MS cortex and in MS NAWM compared to healthy controls (Figure 5C), especially in both, GM and WM lesions (Figure 5C; *P* < 0.05 and *P* < 0.01, respectively). To estimate the extent of Syt7 reduction in axons observed in NAWM and WML, we determined tissue abundance of Syt7 in relation to axonal density. The amount of Syt7 expression correlated positively with the axonal density (Figure 5D; *r* = 0.44, *P* < 0.01). However, the ratio of Syt7 positive area and the axonal density in WML is lower than the corresponding ratio in healthy controls (Figure 5E; WML: *P* < 0.05; NAWM: ns). These results indicate an axonal transport disturbance of Syt7.

**Figure 6:**
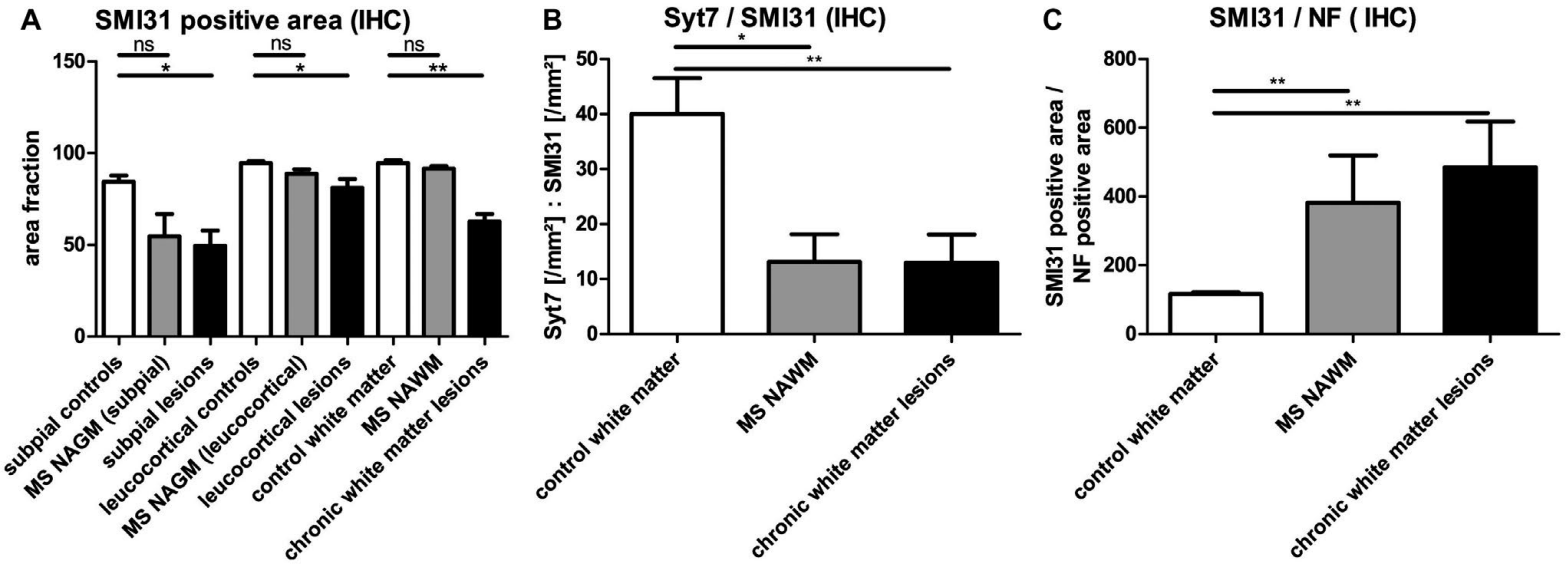
*Potential axonal Syt7 transport deficiency*. **A**. Fewer phosphorylated neurofilaments are present in gray and WM‐lesion areas than in corresponding controls. Relating Syt7‐stained areas to the area of phosphorylated neurofilaments (SMI31 positive) gives a very low quotient for MS NAWM and chronic white matter lesions (**B**). Referencing the immunoreactivity of SMI31 to the immunoreactivity of neurofilaments also gives the impression of hyper‐phosphorylation of the existing axonal structures compared to healthy control tissue (**C**). The SMI31 / Syt7 / Neurofilament stained area was assessed by ImageJ^®^. Mean values are shown + SEM. *P < 0.05; **P < 0.01. The point of reference for the statistical analysis (Mann–Whitney rank sum test) was always the corresponding control tissue of the white or GM of healthy controls (subpial controls: n = 12, MS NAGM (subpial): n = 8, subpial lesions: n = 15, leucocortical controls: n = 12, MS NAGM (leucocortical): n = 10, leucocortical lesions: n = 12, control WM: n = 12, MS NAWM: n = 13, chronic WM lesions: n = 13.

SMI31 is an antibody that stains heavily phosphorylated neurofilament chains 85, which can be used to assess the phosphorylation state of axons. The phosphorylation state of axons has a significant influence on functionality of axonal transport. We found a significant reduction of phosphorylated neurofilaments in lesion sites compared to healthy control tissue (Figure 6A, *P* < 0.05). If the expression of Syt7 is related to the phosphorylated neurofilaments in SMI31 staining (Figure 6B), a significant reduction of Syt7 in phosphorylated axons can be shown both in NAWM and in WM lesions (NAWM: *P* < 0.05; WML: *P* < 0.01). Syt7 reduction in phosphorylated axons is significantly higher than the reduction in all neurofilaments (phosphorylated and unphosphorylated neurofilaments, Figure 5E). Viewing the staining of SMI31 in relation to the staining of pan‐neurofilaments gives the impression that the present axons are hyper‐phosphorylated compared to axons in healthy control tissue. (Figure 6C; *P* < 0.01). These observations underline the idea of a potential axonal transport disorder of Syt7.

Damaged axons also show an enrichment of the dephosphorylated form of the neurofilament heavy chain that can be stained with antibodies against SMI32 86. The number of SMI32‐stained axons was very low in the examined chronic lesions, so the quantitative analysis was inconclusive.

## Discussion

### Syt7 is a miRNA target

In this study, we established the miRNA signatures of gray and white MS lesions and identified seven miRNAs (miR‐let‐7e‐5p, miR‐139‐3p, miR‐4286, miR‐432‐5p, miR‐4488, miR‐574‐5p, miR‐330‐3p) that were upregulated in GM lesions but exhibited no or only low upregulation (or even downregulation) in WM lesions. Our *in‐silico* miRNA target search converged to the neuronal protein Syt7, which had potential binding sites for six of the seven miRNAs mentioned above. The demonstration of Syt7 3′UTR‐miRNA interactions with five miRNA‐binding sites indicates a true interaction of these miRNAs—namely miRs let7e‐5p, miR‐4286, miR‐432‐5p, miR‐4488 and miR‐330‐3p—and the Syt7 transcript *in vivo and in vitro*.

### Syt7 is maldistributed in MS‐tissue

The miRNA‐target Syt7 is expressed mainly in neuroaxonal structures and shows a maldistribution with accumulation in the neuronal cell bodies and simultaneous reduction of the staining intensity in the WM. The lower Syt7 reactivity in axons is partly due to a general reduction of axonal structures (Figure 5C), which has already been described in NAWM areas previously 87. The stronger decrease of the Syt7 staining intensity in WML may indicate an axonal transport disturbance. Another possible factor that might have an influence on the reduced staining intensity is a reduction in the number of oligodendrocytes and their myelin sheaths in WML.

### Axonal Syt7 transport disturbance could be a reason for Syt7 maldistribution

In the literature, there is evidence for an axonal transport disorder in MS. For example, amyloid precursor protein (APP) accumulation was described in damaged axons in lesions 26 and outside demyelinated areas 10, and a reduction of motor proteins such as KIF5A and its transport was shown in WM lesions and NAWM 33. Other neurological diseases including Alzheimer's disease 72, amyotrophic lateral sclerosis 9 and Huntington's disease 48 also exhibit axonal transport disorders that might represent a mechanism leading to neurodegenerative processes 18, 61, 62. Since the intracellular transport system is essential for the development and maintenance of dendritic spines 7, its reduced efficiency can also explain the recent observation of a significant reduction in the number of spines in the cortex of MS patients 38.

The main potential causes of a possible axonal transport disorder in MS would be demyelination, inflammation, decay of mitochondria and glutamate toxicity (reviewed by 8). The consequence of a transport disorder would be an axonal / neuronal retrograde accumulation of protein or organelles (reviewed by 8), which is not necessarily limited to the lesions. Since axons often run through damaged white matter, the corresponding neurons can be affected in projection, so that they do not necessarily have to lie in a lesion area in the cortex. In fact, we have shown Syt7 accumulation in the neuronal soma also independent of lesion areas and in single axons, whereas most of the axons exhibited significantly reduced Syt7 expression. The fact that Syt7 accumulates in neuronal soma of MS patients could be explained by a *distribution disorder* of Syt7 since MS patients’ brains show almost identical numbers of neurons but simultaneous axonal reduction in comparison to brains of healthy controls. However, the observation that Syt7 is reduced in axons of MS‐brains to a greater extent than one might have expected from the density of the axonal scaffold makes an additional axonal *transport disorder* plausible. This hypothesis is supported by the additional significant reduction of Syt7 in the non‐demyelinated NAWM.

Phosphorylation of neurofilaments plays an important role in efficient axonal transport. Neurons and axons contain a variable amount of phosphorylated neurofilaments of different molecular weights—high (Nf‐H), medium (Nf‐M) and low (Nf‐L)—with essential functions in the organization of cytoskeleton, axonal transport and regeneration 44, 81. In chronic lesions, lower levels of phosphorylated SMI31^+^ neurofilaments (Nf‐H, phosphorylated) are detectable 65, 77. In line with this, we observed a decrease in immunoreactivity to SMI31 in both GM and WM lesions. In accordance with two other studies 10, 65 we were unable to demonstrate a quantitative difference in hypo‐phosphorylated Nf‐H levels between lesions and controls using immunohistochemical staining for SMI32 (data not shown), whereas an increase of hypo‐phosphorylated neurofilaments in MS has been described in other studies 28, 29, 66, 86, 98. However, Nf‐H *hyper‐phosphorylation* has been demonstrated in acute lesion stages in relation to retrograde neuronal degeneration and decreasing phosphatase activity in experimental studies in aging mice 65, 76, 89. Furthermore, a stronger increase in the proportion of Nf‐H phosphorylation in spinal tissue compared to cerebral tissue has been demonstrated in MS vs. controls 65. Interestingly, hyper‐phosphorylated neurofilaments were also congested at the margins of chronically inactive lesions 65. Nf‐H phosphorylation and the velocity of axonal transport were inversely correlated 1, 96 suggesting an impairment of the axonal transport machinery due to hyper‐phosphorylation. If the phosphorylated neurofilament in our study is referred to the axonal density (measured with pan‐NF‐antibodies), the impression arises that neurofilament structures in chronically inactive lesions show hyper‐phosphorylation compared to controls. If we now relate Syt7 to SMI31, we show that the Syt7/SMI31 ratio is even lower than the Syt7/NF ratio, which emphasizes the impression of a transport disorder.

### miRNAs are co‐regulated with Syt7

miRNAs recognize partially complementary target sequences in corresponding mRNAs and destabilize their mRNA targets or inhibit protein translation 24, 99. The interaction between miRNAs and Syt7 in neurons could therefore be able to decrease Syt7 (transcript and/or protein). Nonetheless, we found simultaneous accumulation of Syt7 in neurons and upregulation of miRNA expression.

**Figure 7:**
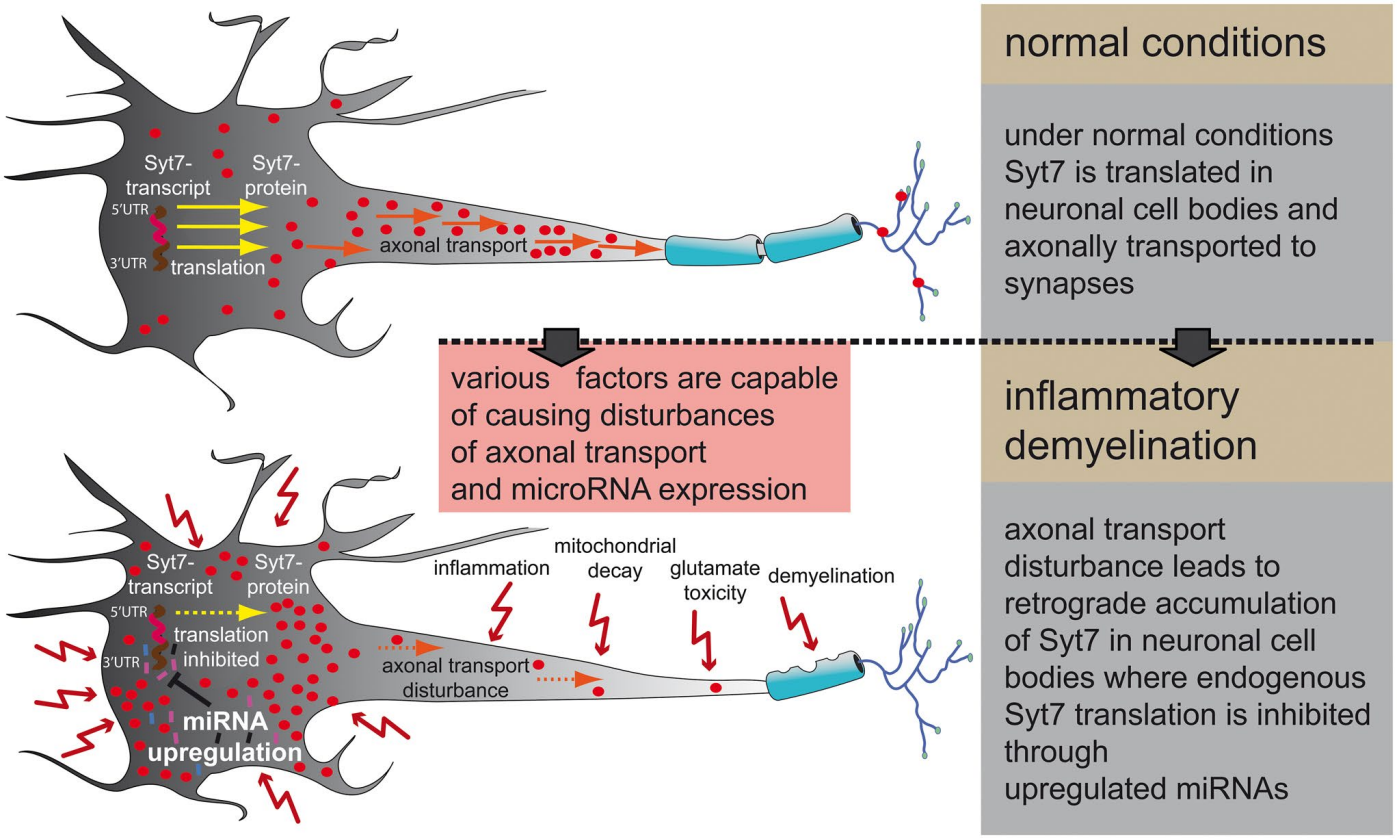
*A possible explanation for the observed Syt7 expression*. Under normal conditions Syt7 is translated in neuronal cell bodies and axonally transported to synapses afterward (upper panel). In MS, various factors are probably able to disturb axonal transport on the one hand and microRNA expression on the other. The disturbance of axonal transport leads to retrograde accumulation of Syt7 in neuronal soma (lower pannel), where endogenous Syt7 translation is inhibited through independently upregulated miRNAs.

At first glance, this does not seem to be consistent. However, since the transcript of Syt7 is not elevated in neurons, an axonal distribution/transport disorder of Syt7 appears plausible as described above. Although expression of the five above mentioned miRNAs seemed not to be altered upon Syt7 accumulation itself, we found their expression to be upregulated. This could have been the result of various factors that are altered in MS lesions such as inflammatory cytokines 56, glutamate exitotoxicity 3 or demyelination 37. Although being upregulated independently from Syt7 accumulation, these miRNAs all are capable of inhibiting further endogenous translation of Syt7 (Figure 2C) and hence could act in a counterregulatory manner (Figure 7). However, the pathomechanism remains to be clarified.

### Syt7 maldistribution and functional consequences in MS

Syt7 is involved in the modulation of synaptic transmission. At the presynaptic terminal, it has been proposed to function as high affinity Ca^2+^ sensors for asynchronous release generation 4, 16, 47, 54, 88. Moreover, in its capacity as a Ca^2+^ sensor, it enables short‐term facilitation 35, 88. In addition, Syt7 has a function in synaptic vesicle replenishment 50. It is being discussed that presynaptic dysfunction causes mental illness and cognitive deficits 17. A distribution disorder of Syt7 might therefore have potential effects on synaptic transmission, which in turn leads to the hypothesis that the cognitive deficits in MS patients, such as impaired recent memory, sustained attention, verbal fluency, conceptual reasoning and visuospatial perception 68 are partly due to synaptic dysfunction caused by maldistribution of Syt7. In addition, a reduced density of synapses has been observed in MS patients 55.

The co‐localization of Syt7 with CNP also shows a Syt7 expression in oligodendrocytes. According to our results, miR‐330‐3p is probably also expressed in oligodendrocytes in addition to its expression in neurons, and can interact with the Syt7 transcript (Figure 2B and 2C). Since Syt7 is also a Ca^2+^ receptor in lysosomal membranes, where it mediates the fusion of lysosomes with plasma membranes 57, miRNA‐mediated downregulation of Syt7 in oligodendrocytes could have effects on de‐ and remyelination processes. Plasma membrane repair is regulated by Ca^2+^‐regulated exocytosis of lysosomes, a process in which Syt7 is significantly involved 69.

### Potential targets of dysregulated miRNAs in GM‐MS‐lesions

The miRNAs (let‐7e‐5p, miR‐330‐3p, miR‐139‐3p, miR‐4286, miR‐432‐5p, miR‐4488, miR‐574‐5p) specifically investigated in our study have already been described in the literature in different contexts. For example, **miR‐let‐7e** is involved in the pathogenesis of murine EAE, ie, the animal model of MS; here, miR‐let‐7e was seen mainly in CD4^+^ T cells and mononuclear cells infiltrating the CNS. miR‐let‐7e expression was strongly correlated with the development of EAE symptoms 31. In addition, miRNAs of the let‐7 family have been described several times in various studies in the context of neurogenesis and axonal guidance 58, 60, 74, 94, 101. MiR‐let‐7 was described in conjunction with neurodegenerative processes 45, 80. Knockdown of miR‐let‐7a appeared to have a neuroprotective effect in cerebral ischemia 95. Furthermore, miRNAs from the let‐7 family were detected in lumbar motor neurons in the EAE animal model 39 along with various other miRNAs especially during the peak of disease. **miR‐330** is thought to have a neuroprotective effect, which was demonstrated in Alzheimer's disease with regard to reduced amyloid beta‐protein production, oxidative stress and mitochondrial dysfunction 102. In another study, miR‐330‐5p was shown to have a competitive effect with Rpph1 on the expression of CDC42 and the associated hippocampal neuron dendritic spine formation 14. In prion‐infected mice, **miR‐320** (see Table 1) was detected in synaptoneurosomes 13. In a study, in 5‐month‐old APP/PS1 mice, it was shown that overexpression of **miR‐574** leads to cognitive impairment via neuritin regulation 49. In several other studies, diverse miRNAs have been detected in synaptoneurosomes 53, 83, what leads to the conclusion that miRNAs play a relevant role in synaptic transmission. In fact, miRNAs play a key role in the regulation of spine morphology and excitability 23, 27, 67, 78, 83. Pre‐miRNAs and an inactive form of the miRNA‐processing enzyme (Dicer) were also observed in the environment of the postsynaptic density. Neuronal stimulation can lead to Ca^2+^‐mediated activation of Dicer, resulting in a local increase in functional mature miRNAs 52, 53.

Since Syt7 is involved in synaptic transmission, altered Syt7 expression may be one of the molecular causes of cognitive changes in the course of the disease.

The influence of an altered Syt7 distribution *in vivo* on synaptic transmission in human MS or in animal MS models was not part of this study and has to be investigated in future studies. Also, the probable underlying axonal transport disorder of Syt7 has to be investigated separately for its effect on synaptic transmission and neuroaxonal degeneration.

## Author Contributions

L.F. and A.J. conceived and designed the experiments; L.F., D.S., K.H., F.M. and A.J. performed the experiments. L.F., S.T.‐H. and A.J. analyzed the data. L.F., S.T.‐H. and A.J. wrote the paper.

## Conflicts of Interest

The authors declare no conflicts of interest.

## Supporting information


**Figure S1** **. Double staining of Syt7‐NF, Syt7‐ Syn, Syt7‐CNP, Syt7‐NeuN, Syt7‐Olig2 and Syt7‐GFAP. **The double immunofluorescence of Syt7 (**A‐C**, red; **D‐E**, green) with pan‐neurofilament (**A**, green), synaptophysin (**B**, green), CNP (**C**, green), NeuN (**D**, red), Olig2 (**E**, red) and GFAP (**F**, red) shows that Syt7 can be found in axonal structures (A), is expressed in the cell soma of neurons (B, D) and is also expressed by single oligodendrocytes (**C, E**), whereas no Syt7 was found in astrocytes (**F**); (scale bar **A,B,D,E,F** = 25µm, **C **= 10µm).
**Figure S2** **. Quantification of neurons and oligodendrocytes. A.** The immunohistochemical Syt7 staining of the MS‐cortex repeatedly shows neurons with different staining intensities for Syt7 (strongly stained neurons ‐ arrowheads, weakly stained neurons ‐ arrows), whereby the weakly stained neurons were not included in the quantification (see computer‐generated mask of Syt7 quantification). **B**. The immunohistochemical quantification of NeuN positive neurons with the corresponding computer‐generated mask for cell counting. C: A representative image of the surrounding zone of a white matter lesion with single positively marked oligodendrocytes in Syt‐7 staining and the corresponding computer‐generated mask for cell counting.

## Data Availability

The data that support the findings of this study are available from the corresponding author upon reasonable request.
