# Supplementary material for: MicroRNA profiles of MS gray matter lesions identify modulators of the synaptic protein synaptotagmin‐7
Source: Brain Pathol. 2019 Nov 17;30(3):524–40. doi: 10.1111/bpa.12800 (PMC8018161; doi:10.1111/bpa.12800)
Supplement: Supplementary file 1 — Figure S1 . Double staining of Syt7‐NF, Syt7‐ Syn, Syt7‐CNP, Syt7‐NeuN, Syt7‐Olig2 and Syt7‐GFAP. The double immunofluorescence of Syt7 (A‐C, red; D‐E, green) with pan‐neurofilament (A, green), synaptophysin (B, green), CNP (C, green), NeuN (D, red), Olig2 (E, red) and GFAP (F, red) shows that Syt7 can be found in axonal structures (A), is expressed in the cell soma of neurons (B, D) and is also expressed by single oligodendrocytes (C, E), whereas no Syt7 was found in astrocytes (F); (scale bar A,B,D,E,F = 25µm, C = 10µm). Figure S2 . Quantification of neurons and oligodendrocytes. A. The immunohistochemical Syt7 staining of the MS‐cortex repeatedly shows neurons with different staining intensities for Syt7 (strongly stained neurons ‐ arrowheads, weakly stained neurons ‐ arrows), whereby the weakly stained neurons were not included in the quantification (see computer‐generated mask of Syt7 quantification). B. The immunohistochemical quantification of NeuN positive neurons with the corresponding computer‐generated mask for cell counting. C: A representative image of the surrounding zone of a white matter lesion with single positively marked oligodendrocytes in Syt‐7 staining and the corresponding computer‐generated mask for cell counting. [file BPA-30-524-s001.docx]

**MiRNA profiles of MS grey matter lesions identify modulators of the synaptic protein Synaptotagmin-7**

# Supplementary material:

| **Supplementary Table 1:** MS autopsy cases and controls | | | | | | |
| --- | --- | --- | --- | --- | --- | --- |
| **Case** | **Age** | | **Sex** | | **Cause of Death** | **Inflammatory Circumstannces Perimortal** |
| **Multiple Sclerosis—autopsy cases** | | | | |  |  |
| **MS-01** | 66 | | female | | cancer metastases in the liver resulting in severe failure of the liver functions | no |
| **MS-02** | 75 | | female | | pneumonia | pneumonia |
| **MS-03** | 68 | | female | | pneumonia | pneumonia |
| **MS-04** | 78 | | female | | stroke | no |
| **MS-05** | 49 | | male | | pneumonia | pneumonia |
| **MS-06** | 55 | | male | | respiratory insufficiency complicating pneumonia and urosepsis | pneumonia and urosepsis |
| **MS-07** | 44 | | male | | aspiration pneumonia | pneumonia |
| **MS-08** | 44 | | male | | multiorgan failure | unkown |
| **MS-09 + MS-10** | 57 | | female | | respiratory failure | (uro)sepsis |
| **MS-11** | 53 | | male | | euthanasia | unknown |
| **MS-12** | 62 | | female | | cachexia and pulmonary insufficiency | no |
| **MS-13 + MS-14** | 56 | | female | | respiratory insufficiency in pneumonia | pneumonia |
| **MS-15** | 54 | | female | | heart failure | unknown |
| **MS-16** | 58 | | male | | terminal renal failure | pneumonia |
| **MS-17** | 63 | | male | | pneumonia | pneumonia |
| **MS-18** | 48 | | female | | respiratory failure | unknown |
| **Controls—autopsy cases** | | | | |  |  |
| CON-01 | | 53 | | female | respiratory failure |  |
| CON-02 | | 54 | | male | aortic dissection |  |
| CON-03 | | 56 | | female | multiorgan failure |  |
| CON-04 | | 55 | | male | multiorgan failure | pneumonia |
| CON-05 | | 58 | | female | heart failure |  |
| CON-06 | | 62 | | male | respiratory failure |  |
| CON-07 | | 66 | | female | multiorgan failure |  |
| CON-08 | | 68 | | female | multiorgan failure |  |
| CON-09 | | 57 | | female | heart failure | pneumonia |
| CON-10 | | 62 | | male | heart failure | peritonitis |
| CON-11 | | 75 | | female | heart failure |  |
| CON-12 | | 77 | | female | respiratory failure | pneumonia |

| **Supplementary table 2:** Antibodies and staining procedures | | | |
| --- | --- | --- | --- |
| **Antigen** | **Company** | **Pre-Treatment** | **Dilution** |
| Syt7,ab121383, rabbit pc | Abcam | Citrate | 1:150 |
| NF, M0762, Clone 2F11, mouse mc | Dako | none | 1:3000 |
| Syn, M7315, Clone DAK-SYNAP, mouse mc | Dako | Citrate | 1:400 |
| CNP, SMI-91R, Clone SMI-91, mouse mc | Covance | Citrate | 1:4000 |
| SMI31, 801601, mouse mc | BioLegend | Tris-EDTA | 1:1000 |
| SMI32, 801701, mouse mc | BioLegend | Citrate | 1:3500 |
| MBP, REF A0623, rabbit pc | DAKO | none | 1:1000 |
| NeuN, ab104225 | Abcam | Citrate | 1:1000 |
| Olig2, REF 18953, rabbit pc | IBL | Tris-EDTA | 1:300 |
| GFAP, REF 0334, rabbit pc | Dako | none | 1:1000 |

| **Supplementary table 3:** Sequences of the 3'UTR of Syt7 used for cloning into the 3’UTR of luciferase | | | | | |
| --- | --- | --- | --- | --- | --- |
| designation of miRNA  binding site | degree of conservation based on  Targetscan 5.0 | miRNA | Position in the 3'UTR  of Syt7 | seed  region | sequence used for cloning^A^ |
| C1- 330-3p | Conserved | miR-330-3p | 225-231 | 7mer-m8 | 5’-ATATAAGCTTGTAAGTCAGGAAGAAAAGGGGCAAAGCAGGAAAATGCCTCCCAGAGCCCCTTCCCCGGAGACTAGTTTAA – 3’ |
| U1-4286 | Poorly conserved | miR-4286 | 575-581 | 7mer-  A1 | 5’-ATATAAGCTTTCCGCATTTCCAGGCCTCTGTCCCCACGCACACACACAATGGACACAAACTAGTTTAA – 3’ |
| U1-139-3p | Poorly conserved | miR-139-3p | 773-779 | 7mer-  m8 | 5’-ATATAAGCTTGGCACCAAGGGGAGAAGGGGAGGAGACGGGGGGATGGCAGGAGGCAGCCACTAGTTTAA -3’ |
| U1-4488 | Poorly conserved | miR-4488 | 1531-1537 | 7mer-  A1 | 5’-ATATAAGCTTAAGACCACGGCCACAGACTGTGGGGGCCTGGCGCCACCTGGCGGTAGTACTAGTTTAA -3’ |
| C1-let-7e-5p | Conserved | miR- let-7e-5p | 2617-2624 | 8mer | 5’-ATATAAGCTTGTGGCCTCACAACCTCCCCTATGAGGTAGGTGAAGTATTATTACCATCATTTTACAGATACTAGTTTAA -3’ |
| U1-432-5p | Poorly conserved | miR-432-5p | 3665-3671 | 7mer-  m8 | 5’-ATATAAGCTTCCTTTGGTTTGTCTGAGCTGCCTTGGAGGGTGGCAAGTGTCCTCTGGGAACTAGTTTAA -3’ |
| ^A^ double stranded oligonucleotides were inserted in the predesigned vector; the sequence of one strand 5'->3' is given | | | | | |

| **Supplementary table 4:** miRNA-expression in white matter MS lesions and normal appearing white matter | | | | | | |
| --- | --- | --- | --- | --- | --- | --- |
| **miRNAs**  **up-regulated**  **in lesions / tissue^a^** | **Percent surrogate**  **housekeeping gene^b^**  **in lesions / tissue** | **Fold regulation in lesions /tissue compared to normal brain WM^c^** |  | **miRNAs**  **down-regulated**  **in lesions tissue^a^** | **Percent surrogate**  **housekeeping gene^b^ in lesions /tissue** | **Fold regulation in lesions compared to normal brain WM^c^** |
| **miRNA profiles in inactive WM lesions** | | | | | | |
| miR-1285-5p | 171.9 | 36.9** |  | miR-330-3p^e^ | 0.2 | 0.03** |
| miR-2682-5p | 6.9 | 31.7* |  | miR-219a-2-3p | 40.7 | 0.08** |
| miR-574-5p | 57.5 | 21.8* |  | miR-181a-3p | 0.7 | 0.09** |
| miR-122-5p | 57.4 | 17.2* |  | miR-219a-5p^e^ | 56.3 | 0.12** |
| miR-888-5p | 23.0 | 15.3* |  | miR-194-5p | 0.3 | 0.14** |
| miR-3065-5p | 25.8 | 15.1** |  | miR-197-3p | 0.6 | 0.14** |
| miR-3144-3p | 17.0 | 10.5** |  | miR-133a-3p | 0.2 | 0.16** |
| miR-1260a | 94.4 | 10.5* |  | miR-151a-5p^e^ | 5.1 | 0.16** |
| miR-1972 | 117.8 | 10.4* |  | miR-181a-5p | 148.7 | 0.18** |
| miR-612 | 14.2 | 9.7** |  | miR-328-3p | 0.3 | 0.18** |
| miR-92b-3p | 11.2 | 7.8* |  | miR-574-3p | 1.2 | 0.18** |
| miR-155-5p | 7.7 | 7.2** |  | miR-425-5p | 0.5 | 0.19** |
| miR-497-5p^d^ | 77.6 | 7.0** |  | miR-33a-5p | 0.8 | 0.23** |
| miR-4454+ | 12350.1 | 6.7** |  | miR-18a-5p^e^ | 0.4 | 0.24** |
| miR-7975 |  |  |  | miR-190a-5p^e^ | 2.0 | 0.25** |
| miR-195-5p^d^ | 17.8 | 5.5** |  | miR-181b-5p+^e^ | 5.9 | 0.28** |
| miR-135a-5p^d^ | 65.6 | 5.1** |  | miR-181d-5p^e^ |  |  |
| miR-30a-3p^d^ | 8.8 | 4.8* |  | miR-337-5p | 0.2 | 0.34* |
| miR-1180-3p | 72.5 | 4.5** |  | miR-181c-5p | 9.2 | 0.35** |
| miR-23a-3p^d^ | 336.6 | 4.3** |  | miR-154-5p | 1.1 | 0.39* |
| miR-130a-3p^d^ | 100.9 | 3.9** |  | miR-23b-3p^e^ | 52.6 | 0.39** |
| miR-378i | 6.4 | 3.8* |  | miR-340-5p^e^ | 20.5 | 0.40** |
| miR-148a-3p^d^ | 12.3 | 3.6* |  | miR-885-5p | 5.1 | 0.49* |
| miR-4286 | 6845.0 | 3.5* |  |  |  |  |
| miR-99a-5p | 411.8 | 3.3** |  |  |  |  |
| miR-30a-5p^d^ | 41.9 | 3.2** |  |  |  |  |
| miR-664a-3p | 13.3 | 3.1* |  |  |  |  |
| let-7c-5p^d^ | 367.6 | 2.9** |  |  |  |  |
| miR-204-5p^d^ | 27.2 | 2.8* |  |  |  |  |
| miR-34a-5p | 52.7 | 2.7* |  |  |  |  |
| miR-30e-3p | 5.8 | 2.7* |  |  |  |  |
| miR-30d-5p^d^ | 46.2 | 2.6** |  |  |  |  |
| miR-142-3p | 83.9 | 2.6** |  |  |  |  |
| miR-362-5p | 2.3 | 2.6* |  |  |  |  |
| miR-150-5p | 65.5 | 2.3* |  |  |  |  |
| miR-9-5p^d^ | 1375.9 | 2.3** |  |  |  |  |
|  |  |  |  |  |  |  |
| **miRNA profiles in MS NAWM** | | | | | | |
| miR-151a-5p | 18.7 | 3.7* |  | miR-504-5p | 0.0 | 0.03** |
| miR-133a-3p | 0.5 | 2.3* |  | miR-656-3p | 0.2 | 0.12* |
| miR-574-3p | 2.8 | 2.3** |  | miR-491-5p | 0.1 | 0.13* |
| miR-194-5p | 0.6 | 2.2** |  | miR-630 | 8.4 | 0.20* |
|  |  |  |  | miR-2682-5p | 1.7 | 0.25** |
|  |  |  |  | miR-1260a | 27.6 | 0.29** |
|  |  |  |  | miR-660-5p | 0.5 | 0.37** |
|  |  |  |  | miR-30b-5p | 4.5 | 0.38* |
|  |  |  |  | miR-30c-5p | 6.3 | 0.40* |
|  |  |  |  | miR-574-5p | 23.0 | 0.40* |
|  |  |  |  | miR-381-3p | 0.6 | 0.44** |
|  |  |  |  | let-7c-5p | 161.4 | 0.44* |
|  |  |  |  | miR-377-3p | 1.6 | 0.45* |
|  |  |  |  | miR-126-3p | 75.9 | 0.46* |
|  |  |  |  | miR-487b-3p | 2.3 | 0.46** |
|  |  |  |  | miR-122-5p | 26.5 | 0.46* |
|  |  |  |  | miR-129-2-3p | 10.1 | 0.47* |
|  |  |  |  | miR-3065-5p | 12.1 | 0.47** |
|  |  |  |  | miR-186-5p | 1.3 | 0.49* |
| a The miRNAs listed were significantly up-regulated by at least two-fold (**P<0.01,*P<0.05; U-test) in inactive lesions or MS NAWM compared to normal brain.  b Surrogate housekeeping gene: the median of the 36 most abundant miRNAs (median copy number >800).  c Seven inactive lesions, seven MS NAWM specimens and eleven control WM specimens were examined.  d microRNAs which were previously described as up-regulated in chronic inactive WM MS lesions [23].  e microRNAs which were previously described as down-regulated in chronic inactive WM MS lesions [23]. | | | | | | |


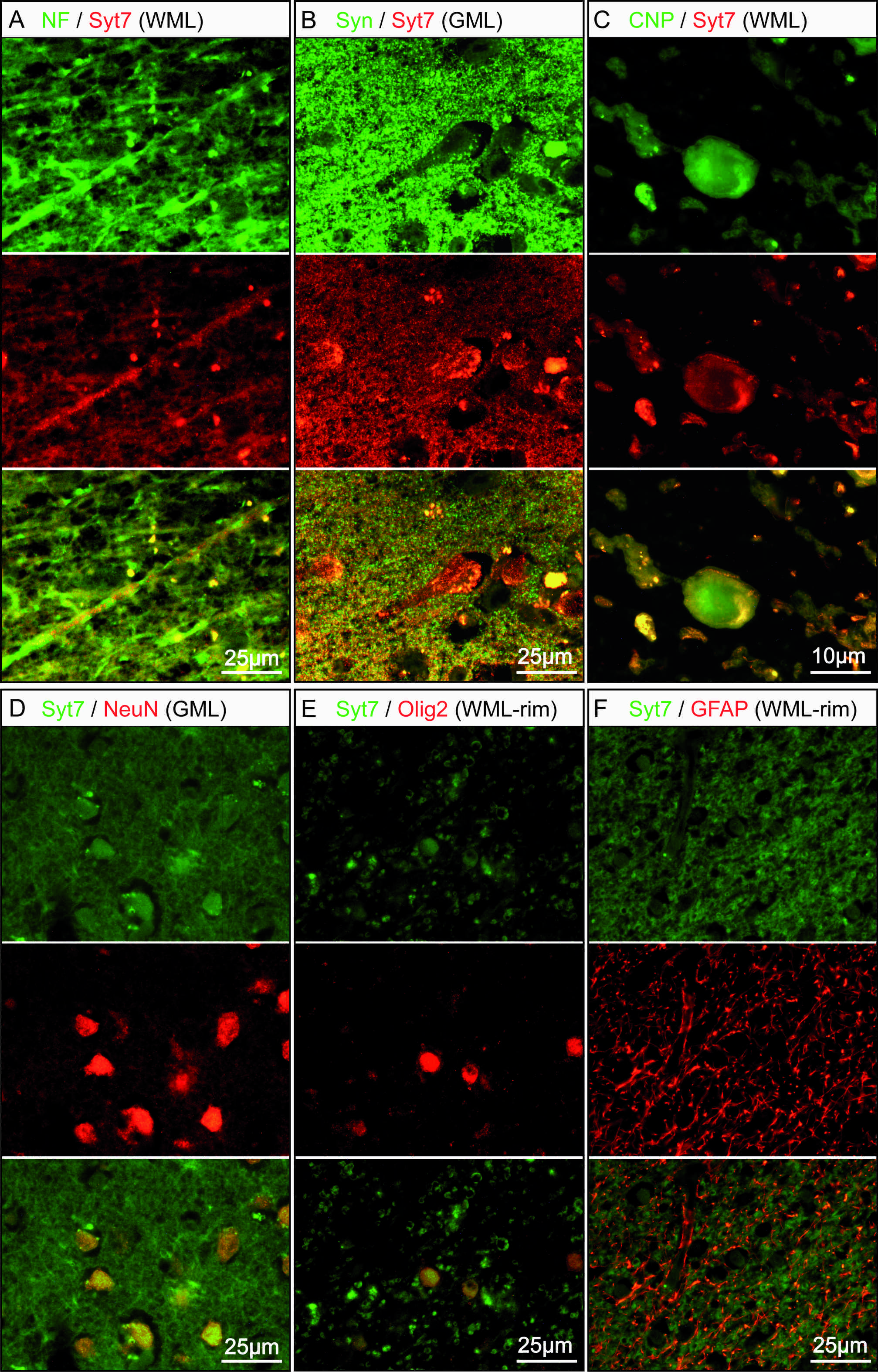


**Supplementary Fig. 1: Double staining of Syt7-NF, Syt7- Syn, Syt7-CNP, Syt7-NeuN, Syt7-Olig2 and Syt7-GFAP.**

The double immunofluorescence of Syt7 (A-C, red; D-E, green) with pan-neurofilament (A, green), synaptophysin (B, green), CNP (C, green), NeuN (D, red), Olig2 (E, red) and GFAP (F, red) shows that Syt7 can be found in axonal structures (A), is expressed in the cell soma of neurons (B, D) and is also expressed by single oligodendrocytes (C, E), whereas no Syt7 was found in astrocytes (F); (scale bar A,B,D,E,F = 25µm, C=10µm).


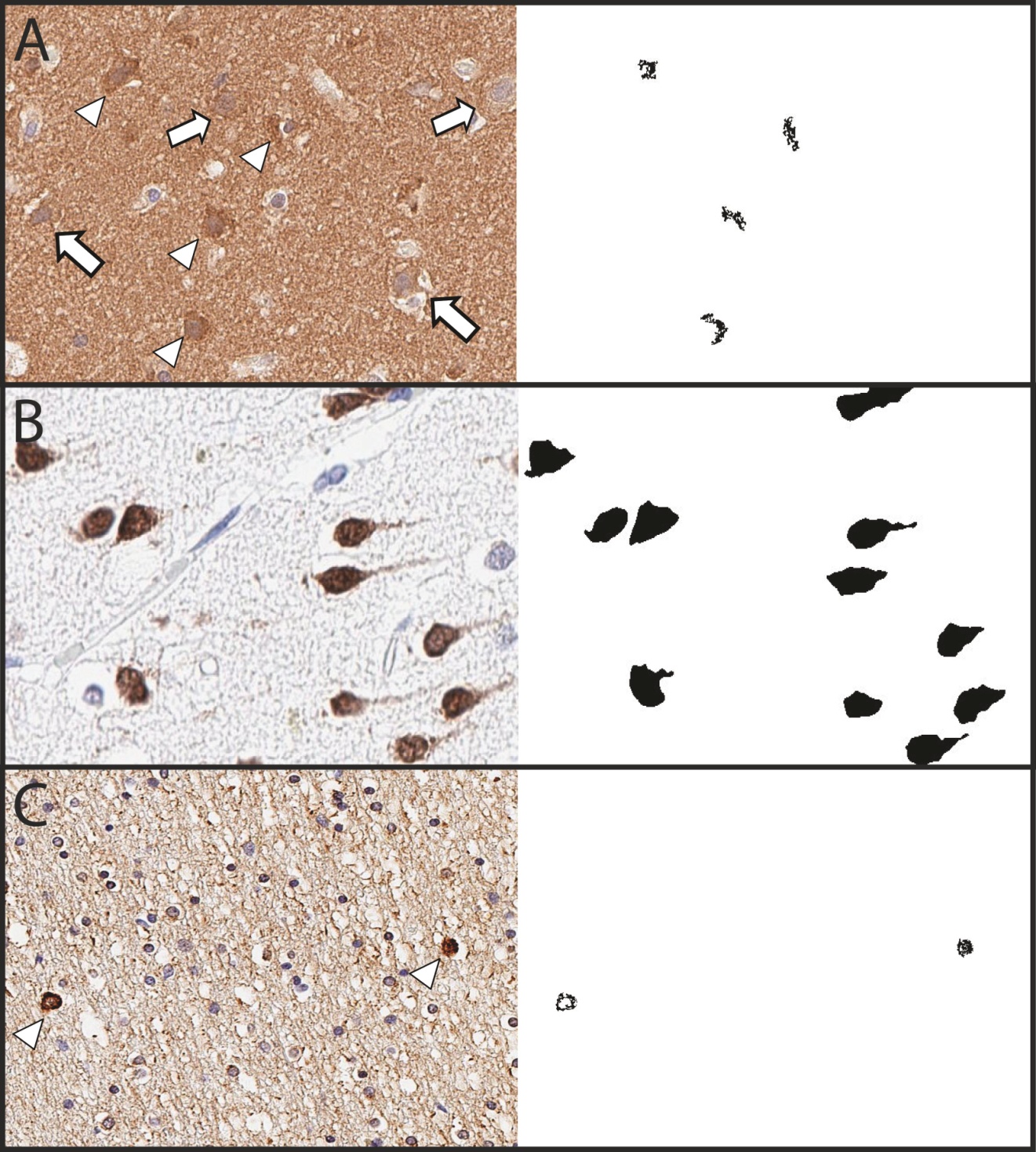


**Supplementary Figure 2: Quantification of neurons and oligodendrocytes**

A: The immunohistochemical Syt7 staining of the MS-cortex repeatedly shows neurons with different staining intensities for Syt7 (strongly stained neurons - arrowheads, weakly stained neurons - arrows), whereby the weakly stained neurons were not included in the quantification (see computer-generated mask of Syt7 quantification).

B: The immunohistochemical quantification of NeuN positive neurons with the corresponding computer-generated mask for cell counting.

C: A representative image of the surrounding zone of a white matter lesion with single positively marked oligodendrocytes in Syt-7 staining and the corresponding computer-generated mask for cell counting.
